# Rapid recovery of soil bacterial communities after wildfire in a Chinese boreal forest

**DOI:** 10.1038/srep03829

**Published:** 2014-01-23

**Authors:** Xingjia Xiang, Yu Shi, Jian Yang, Jianjian Kong, Xiangui Lin, Huayong Zhang, Jun Zeng, Haiyan Chu

**Affiliations:** 1 State Key Laboratory of Soil and Sustainable Agriculture, Institute of Soil Science, Chinese Academy of Sciences, East Beijing Road 71, Nanjing 210008, China; 2 State Key Laboratory of Forest and Soil Ecology, Institute of Applied Ecology, Chinese Academy of Sciences, Wenhua Road 72, Shenyang 110164, China; 3 University of the Chinese Academy of Sciences, Beijing 100049, China; 4 These authors contributed equally to this work.

## Abstract

Fires affect hundreds of millions of hectares annually. Above-ground community composition and diversity after fire have been studied extensively, but effects of fire on soil bacterial communities remain largely unexamined despite the central role of bacteria in ecosystem recovery and functioning. We investigated responses of bacterial community to forest fire in the Greater Khingan Mountains, China, using tagged pyrosequencing. Fire altered soil bacterial community composition substantially and high-intensity fire significantly decreased bacterial diversity 1-year-after-burn site. Bacterial community composition and diversity returned to similar levels as observed in controls (no fire) after 11 years. The understory vegetation community typically takes 20–100 years to reach pre-fire states in boreal forest, so our results suggest that soil bacteria could recover much faster than plant communities. Finally, soil bacterial community composition significantly co-varied with soil pH, moisture content, NH_4_^+^ content and carbon/nitrogen ratio (P < 0.05 in all cases) in wildfire-perturbed soils, suggesting that fire could indirectly affect bacterial communities by altering soil edaphic properties.

Fire is one of the most critical threats to forest ecosystems^1^,^2^,^3^ and its overall effects are complex, ranging from the removal of aboveground biomass to altering the physical, chemical and biological components of soil ecosystems^2^,^4^. Fire produces a broad spectrum of effects that depend on fuel load and combustion, vegetation type, climate, topography and so on. Changes in the type, intensity, frequency and timing of fire disturbance^3^,^5^ due to climate change and human influence may degrade ecosystem function and diversity or perhaps shift the ecosystem to another state.

Despite the essential role that bacteria play in ecosystem recovery^6^, there is little information about the long-term effects of fire on bacterial communities in soils. Fires likely have large direct and indirect effects on soil bacterial community composition and diversity. Heat from fires can kill soil bacteria, reducing microbial biomass^7^ and directly impacting bacterial community composition and diversity^6^. Microbes differ in sensitivity to fire-induced heat^8^: bacteria tend to be more resistant to heat than fungi, and generally increase in relative abundance after a fire. The rapid capacity to re-colonize soil can be decisive in determining post-fire microbial community structure^9^. Indirect effects of fire include changes to many soil properties, including consuming organic material and changing soil chemical properties^10^,^11^. Most fires result in overall losses of soil C and N, but a pulse of ammonium (inorganic NH_4_^+^) often follows fires^4^,^7^. Fires can increase N availability to bacteria because of reduced plant uptake and enhanced mineralization^10^,^11^. Fires can also create a reactive charcoal layer that affects soil available nitrogen and pH^12^,^13^,^14^. Additionally, severe fires often alter forest canopy, litter layer, and soil permeability, thereby influencing soil moisture content, temperature, and pH^15^,^16^. Impacts of fire on bacterial growth and activity can persist for many years after burning^17^, although little research has gone into understanding the long-term effect of fire on soil bacteria.

The Greater Khingan Mountains harbor the largest forest in China. Forest fire has been an increasingly common phenomenon in the Greater Khingan Mountains region in recent decades. From 1965 to 2009, there were 1,552 fires, and the fire interference area was about 66,000 km^2^ in the Greater Khingan Mountains^18^. The objectives of this study were to examine changes in soil bacterial community composition and diversity 1 (short-term) and 11 years (long-term) after the occurrence of forest fire, and to correlate these shifts to edaphic properties. Our work was focused on studying the response of the soil bacteria to wildfire disturbance, emphasizing the main factors driving soil bacterial community composition and diversity after fire, which may improve understanding of post-fire forest ecosystem recovery process.

## Results

### Effects of fire on soil biogeochemical properties

Fire significantly altered soil biogeochemical properties (Table S1). Burning reduced microbial biomass carbon and nitrogen, with 82% less microbial biomass carbon and 71% less microbial biomass nitrogen in 1-year-post-fire burned soils than unburned samples, and 63% less microbial biomass carbon and 72% less microbial biomass nitrogen in 11-year-post-fire burned soils. Wildfire increased soil pH, available nitrogen and phosphorus, and decreased soil moisture and carbon/nitrogen ratio 1-year-post-fire, but after 11 years, those properties were not significantly different from the levels observed in the unburned control site.

### Bacterial community composition

Across all soil samples, we obtained a total of 319,618 quality sequences with 4,123–9,130 sequences per sample (mean 5,327), and were able to classify 84.3% of those sequences. The dominant phyla (or subphyla in the case of *Proteobacteria*) across the Greater Khingan Mountains soils were *Alphaproteobacteria, Actinobacteria, Acidobacteria, Betaproteobacteria* and *Bacteroidetes*, accounting for more than 76% of the bacterial sequences from each of the soils (Fig. S1). In addition, *Gammaproteobacteria, Planctomycetes, Chloroflexi, Deltaproteobacteria, Gemmatimonadetes* and *Firmicutes* were present in most soils but at relatively low abundances, and 24 other rare phyla were identified (Table S2).

**Figure 1:**
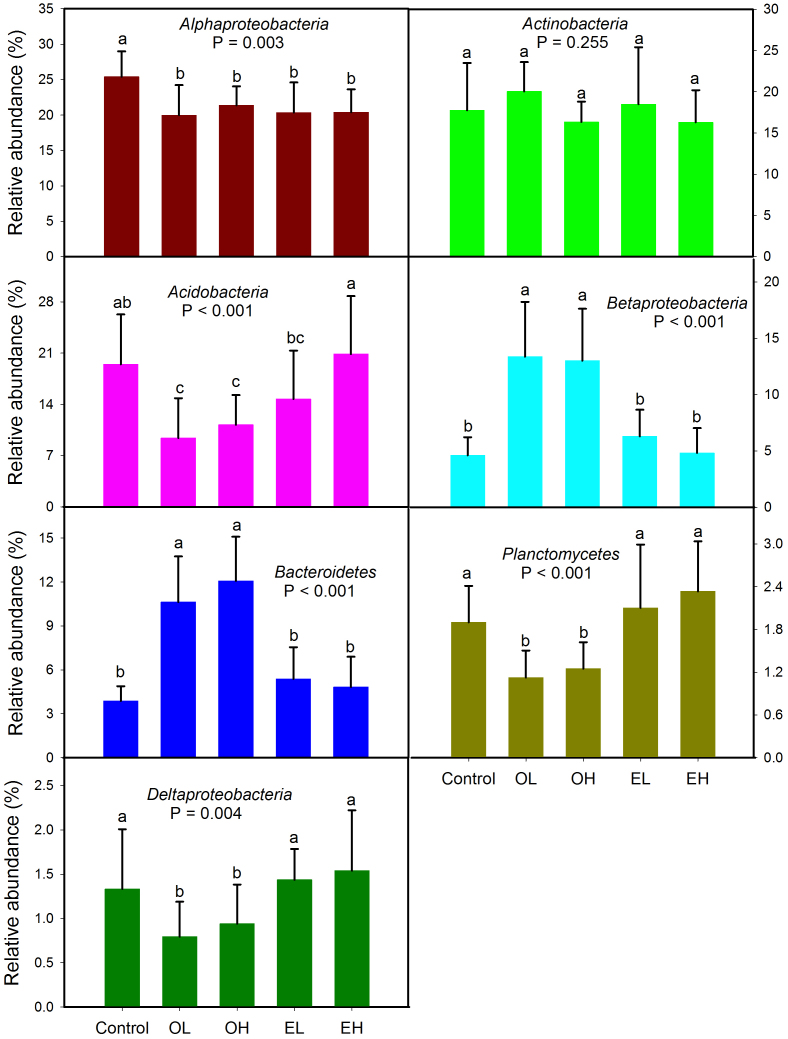
The relative abundances of the dominant bacterial phyla in control and post-fire soils. Error bars denote standard deviation; different letters represent significant differences from Tukey's HSD comparisons (P < 0.05). OL: one year after low intensity fire; OH: one year after high intensity fire; EL: 11 years after low intensity fire; EH: 11 years after high intensity fire.

**Figure 2:**
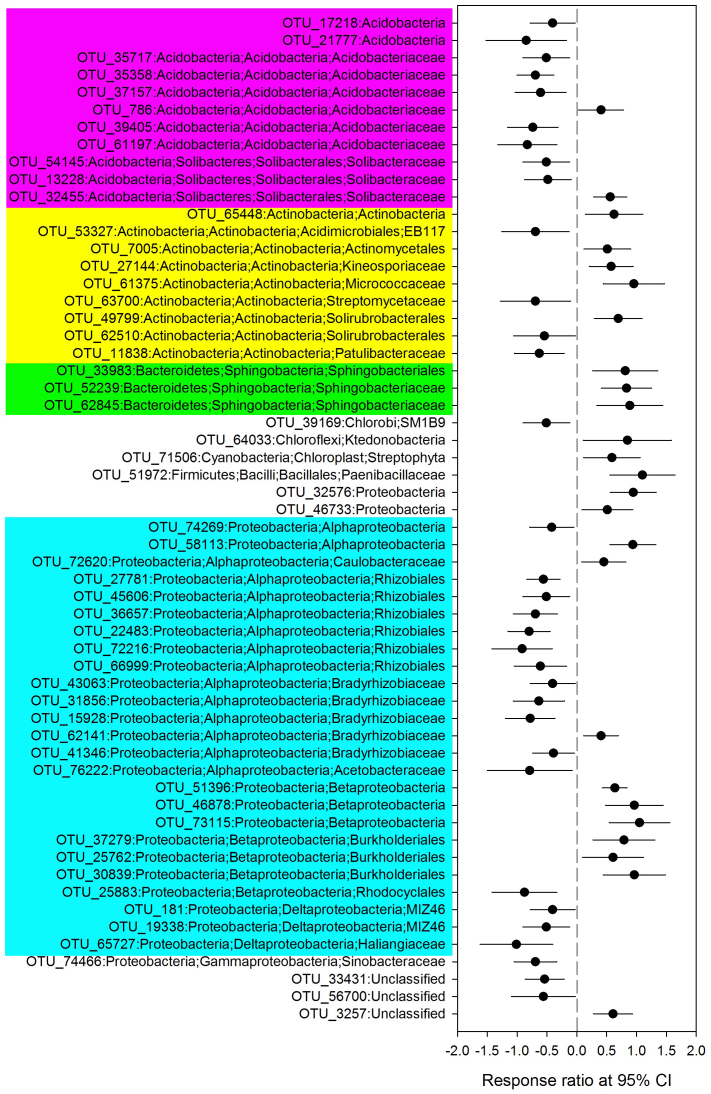
OTUs that exhibited significant changes in abundance at 1 year after high intensity fire. Significance was determined using response ratio methods at a 95% CI (confidence interval).

**Figure 3:**
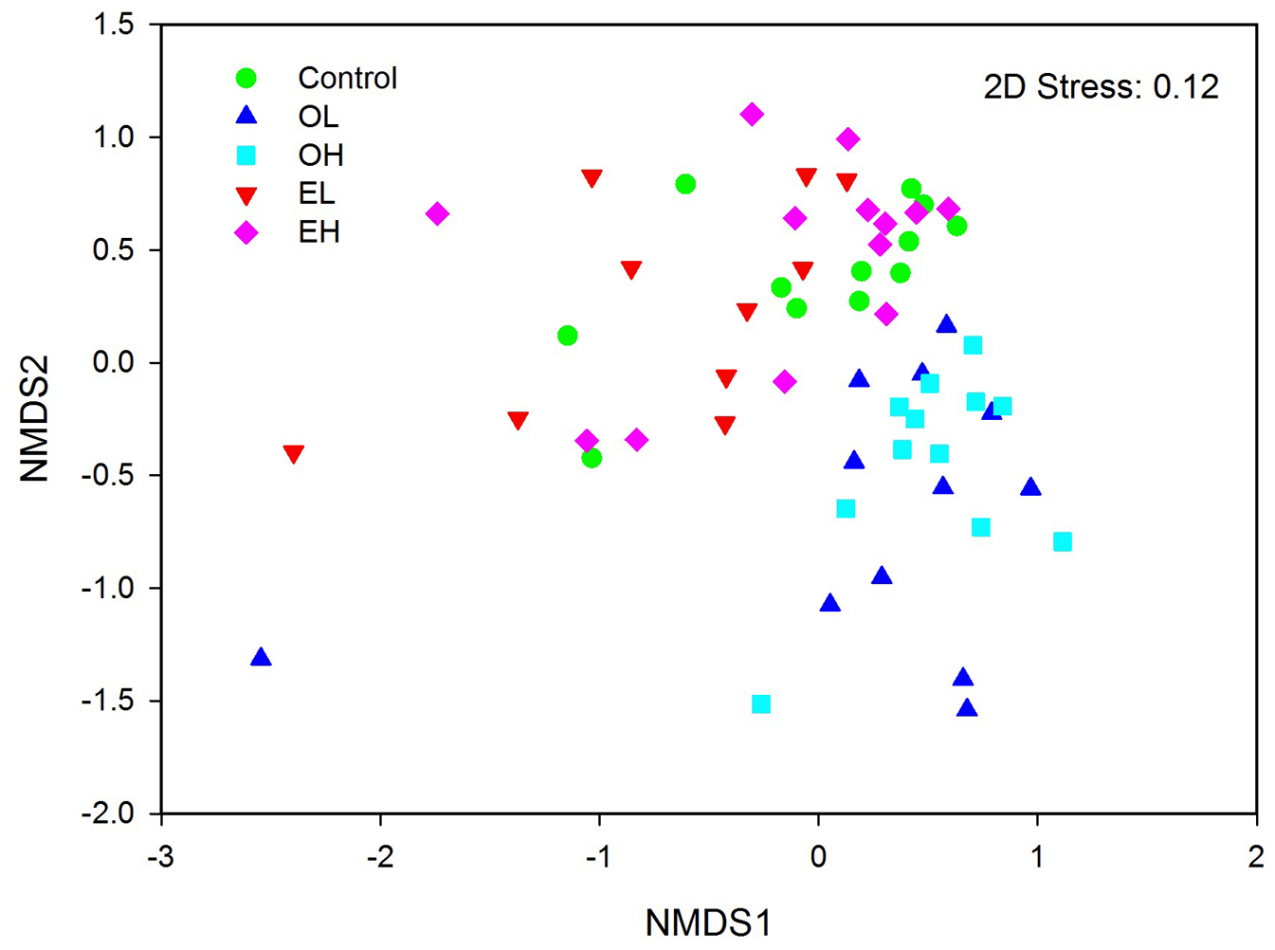
Bacterial community compositional structure in soils across the Greater Khingan Mountains indicated by non-metric multi-dimensional scaling (NMDS) using Bray-Curtis dissimilarity. OL: one year after low intensity fire; OH: one year after high intensity fire; EL: 11 years after low intensity fire; EH: 11 years after high intensity fire.

Fire significantly shifted the relative abundance of dominant phyla except *Actinobacteria* (Fig. 1). Fire greatly increased the relative abundances of *Betaproteobacteria* and *Bacteroidetes* and decreased the abundance of *Alphaproteobacteria*, *Acidobacteria*, as well as *Planctomycetes* and *Deltaproteobacteria* with low abundances 1-year-post-fire. Interestingly, the relative abundances of dominant phyla returned to a similar level to the controls after 11 years, with the exception of *Alphaproteobacteria* (Fig. 1). However, by comparing OH (1 year post high intensity fire) with control at class or lower levels certain taxa demonstrated significant responses. In the phylum *Acidobacteria*, all OTUs (operational taxonomic unit) had a significantly lower abundance except OTU_786 and OTU_32455 in response to fire, and for *Bacteroidetes*, in the family *Sphingobacteria*, all OTUs had a significantly higher abundance (Fig. 2). In the phylum *Proteobacteria, Alphaproteobacteria* demonstrated significantly lower abundance, except for OTU_62141, OTU_58113 and OTU_72620; *Betaproteobacteria* had a significantly greater abundance, except for OTU_25883; while *Deltaproteobacteria* showed lower abundance in response to fire (Fig. 2). In contrast, *Actinobacteria* taxa showed a highly variable response (Fig. 2). Similar patterns were observed when comparing OL (1 year post low intensity fire) with the unburned controls (Fig. S2). Bacterial community composition in soils across the Greater Khingan Mountains showed that fire resulted in a dramatic shift in soil bacterial communities 1-year-post-fire (P = 0.001), but after 11 years the communities were indistinguishable from unburned forest soil (P = 0.549, Fig. 3, Table S3). Fire intensity had no significant impact upon recovery (Fig. 3).

The soil bacterial community was related to soil biogeochemical variables in both pre- and post-fire soils. Mantel tests showed that bacterial community composition was significantly correlated with soil pH in control soils, while the community composition was significantly correlated with soil pH, moisture content, NH_4_^+^ content, and C/N ratio in soils 1 and 11 years post fire (Table 1). Among all the measured soil variables, soil pH showed the highest correlation with bacterial community composition in both control and fire-impacted soils (Table 1). Canonical correspondence analysis (CCA) indicated that soil pH had the strongest effect on bacterial community composition, while soil moisture content, NH_4_^+^ content, C/N ratio and TN content also had less, but significant effect on the community composition (Fig. S3). In addition, soil pH showed a significant correlation with the relative abundance of *Alphaproteobacteria, Actinobacteria, Acidobacteria, Betaproteobacteria*, as well as three less abundant phyla (Fig. S4). Soil NH_4_^+^ content, C/N ratio, moisture content, TC content and TN content were also significantly correlated with the relative abundance of different dominant phyla (Table S4).

### Bacterial diversity

**Figure 4:**
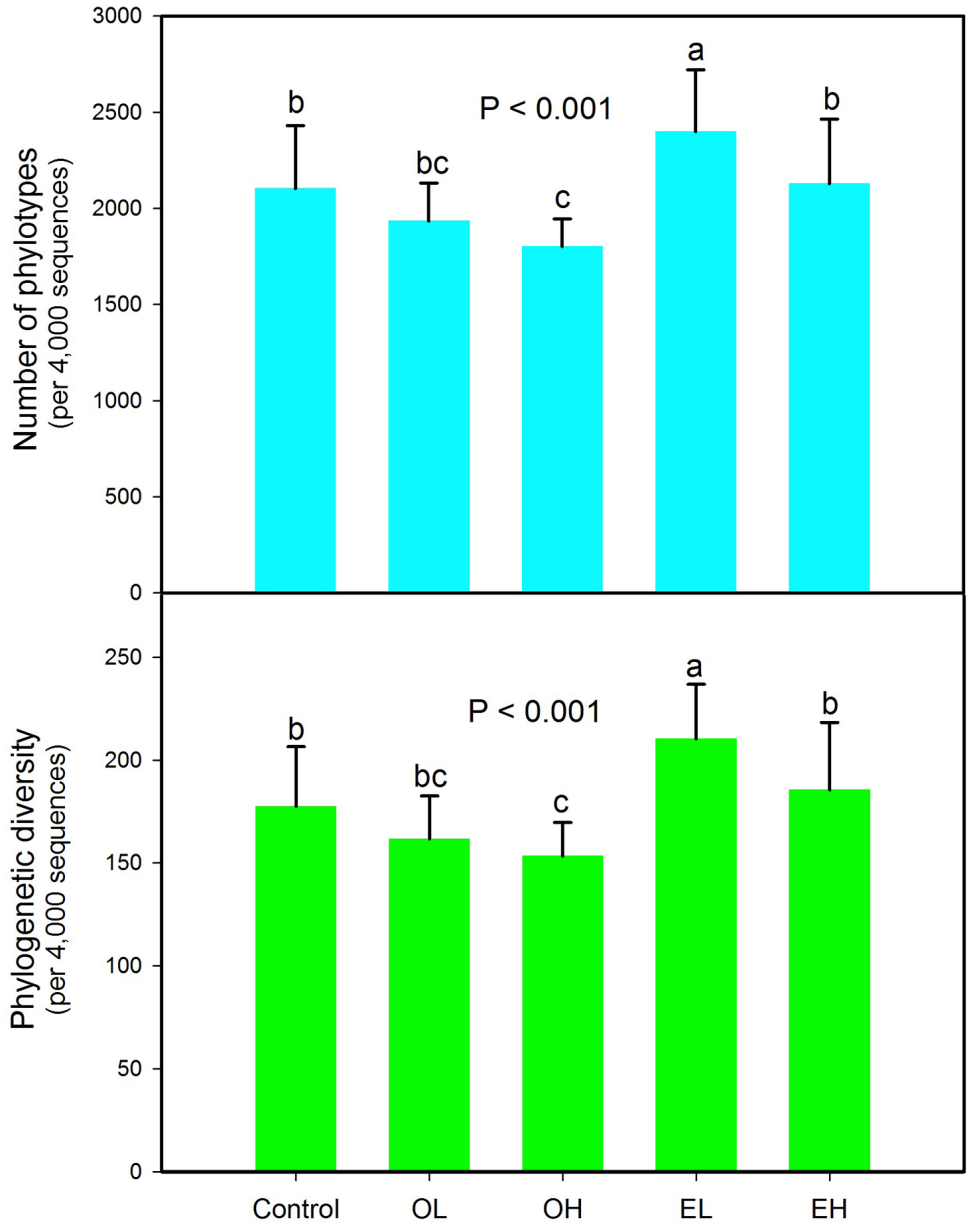
Changes in bacterial OTUs phylotype richness and phylogenetic diversity across the different groups. Diversity indices were calculated using random selections of 4,000 sequences per soil sample. Error bars denote standard deviation; Different letters represent significant differences from Tukey's HSD comparisons (P < 0.05). OL: one year after low intensity fire; OH: one year after high intensity fire; EL: 11 years after low intensity fire; EH: 11 years after high intensity fire.

**Figure 5:**
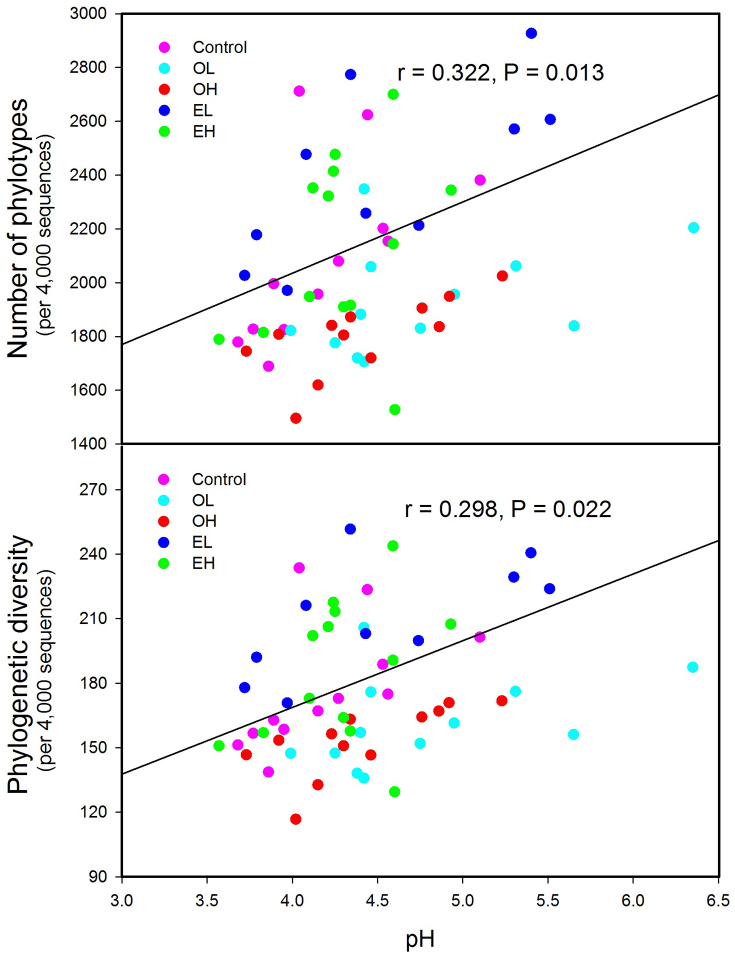
The relationship between soil pH and bacterial OTUs phylotype richness and phylogenetic diversity by linear regression analyses. The communities were randomly sampled at the 4,000 sequences level. Individual points represent different samples across all the treatments. OL: one year after low intensity fire; OH: one year after high intensity fire; EL: 11 years after low intensity fire; EH: 11 years after high intensity fire. P < 0.05, significant convention.

In terms of both phylotype richness (i.e. number of OTUs) and phylogenetic diversity (Fig. 4), which were surveyed at a depth of 4,000 randomly selected sequences per sample, the diversity of bacterial communities exhibited significant differences (P < 0.001 in both cases). High intensity fire quickly decreased bacterial phylotype richness and phylogenetic diversity, but after 11 years, these parameters returned to match the unburned controls. Interestingly, the highest bacterial diversity was found 11 years after low intensity fire. Soil bacterial phylotype richness was positively correlated with soil pH (P = 0.013, Fig. 5), dissolved organic carbon (P = 0.039, Table S5), and negatively correlated with elevation (P = 0.042, Table S5), while phylogenetic diversity was positively correlated with soil pH (P = 0.022, Fig. 5) and dissolved organic carbon (P = 0.049, Table S5).

## Discussion

Records of wildfire occurrences provided an opportunity to examine the effects of fire on soil bacterial community composition, diversity and succession. In this study, we found *Proteobacteria, Actinobacteria, Acidobacteria* and *Bacteroidetes* were the main phyla in boreal forest soil (Fig. S1), similar to observations from other soils collected from Arctic and subalpine soil environments^19^,^20^, showing that dominant bacterial phyla in soils are similar. We found that burning had a dramatic impact on the soil bacterial community composition and diversity 1 year following a fire (Fig. 1, 3, 4). Bacterial communities from 1 and 11 years post-burn were significantly different not only in the OTUs present, but also in the proportional abundances of phyla (Fig. 1, 4, S1; Table S2). An earlier study^21^ found that fire dramatically altered soil bacterial community composition and diversity 4 and 16 weeks after fire and our results showed that the effect of fire on bacterial community could last for more than 1 year, suggesting that fire had a strong impact on bacterial community. Our results go beyond these findings by showing that the composition of bacterial community 11 years after fire returned to a similar state compared to the unburned control site (Fig. 3, Table S3). In addition, bacterial diversity decreased 1-year-post-fire but the diversity recovered 11-year-post-fire with the highest diversity at low intensity fire (Fig. 4), which might be due to the successful colonization and survival of many rare phyla into soil during the process of bacterial succession post fire. Other studies have shown that the post-fire understory vegetation community reaches its pre-fire level in boreal forest 20–100 years after a fire, depending on pre-fire stand age and site conditions^22^,^23^. Our results therefore suggest that bacterial communities may recover much faster than understory vegetation. In the present study, we have only two time points after fire occurrence (1 and 11 years post fire), and in future study more time points post fire might be needed to clarify when the bacterial communities recover to the unburned level and how bacterial communities succeed after forest fire. Human influence and global warming are rapidly increasing the frequency of forest fire, therefore understanding different recovery rate between soil microbial community and vegetation community in response to wildfire may be important for understanding the recovery of forest ecosystems as a whole.

The results in this study showed that bacterial community composition and diversity were mainly correlated with soil pH. The relative abundance of dominant phyla was also correlated with soil pH. For example, the relative abundance of *Acidobacteria* has been shown to increase with decreased pH (Table S4), which is consistent with most of previous studies^20^,^24^. However, the relative abundance of *Alphaproteobacteria* was shown to decrease toward higher pH in our study (Table S4), which is contrary to other studies in different systems^19^,^25^. These results indicated that although bacterial community composition was clearly influenced by pH, there were some differences in the responses of specific phylum to changes in soil pH. The overriding importance of soil pH has been demonstrated as a key factor in driving soil bacterial distribution across a variety of spatial scales, including continents^19^,^26^, national^24^, land-use types at a given location^27^, small and sub-meter scales^28^, and even along elevational gradient^20^. In this study, we observed that the bacterial community composition and diversity were primarily correlated with soil pH in both control and post fire soils (Fig S3; Table 1), suggesting that pH might have predictive power for bacterial distribution in not only undisturbed but also recently fire-perturbed ecosystems.

As noted above, disturbance may trigger both direct and indirect effects on soil microbial community structure. We found that wildfire altered soil pH, moisture content, NH_4_^+^ content and C/N ratio which were significantly co-varied with bacterial community composition in soils both 1 and 11 years after fire (Table 1). These results might suggest that fire could indirectly affect bacterial communities by altering soil properties. In addition, the significance of fire as a shaper of vegetation composition and structure is well known. Major links between plant species and soil microorganisms include the quantity of resources produced, competition for nutrients, quality of resources and mutualism^6^. How the successional growth of vegetation after a wildfire will influence and possibly be influenced by soil microbial community structure is a topic warranting future investigation in this and other study systems.

## Methods

### Site selection and soil sampling

Our study area was located in the Greater Khingan Mountains in northeast China (51°17′N 122°42′E to 51°56′N 123°18′E), and encompassed approximately 167,213 ha. The area has a cold, continental climate, with average annual temperature declining from 1°C at its southern extremes to −6°C at its northern extremes, and precipitation declining from 442 mm in the south to 240 mm in the north. More than 60% of the annual precipitation falls in the summer season from June to August^18^. The vegetation of this area is representative of cool temperate coniferous forests, forming the southern extension of the eastern Siberian boreal forests. Historically, fires were caused primarily by lightning^18^. Dendrochronological studies have indicated that the historical fire regime was characterized by frequent surface fires, mixed with infrequent stand-replacing fires, with the interval between fires ranging from 30 to 120 years^18^,^29^. However, forest harvesting and fire suppression have altered fire regimes in this region^30^.

Soil samples were collected on July 24^th^ to August 19^th^ of 2011 in the Huzhong National Natural Reserve of the Greater Khingan Mountains. The study area is primarily covered by mature larch (Larix gmelinii) forest with little human disturbances since the establishment of the Reserve^29^. The parent material is granite bedrock and the soil is a dark brown forest soil^31^. We used a stratified sampling design to select sample plots based on fire history and fire severity. Fire history (surface fire) has three levels: 1-year-after-fire, 11-year-after-fire and unburned control. Fire severity was defined into two levels: low and high severities. Fire severity levels were defined based on differenced normalized burn ratio (dNBR) of remote sensing Landsat TM images, which have been proved applicable to our study area^29^. The dNBR^32^,^33^ was calculated using the equation NBR_pre-fire_ − NBR_post-fire_, while NBR was calculated using the equation (TM4 − TM7)/(TM4 + TM7). TM4 and TM7 refer to Thematic Mapper bands 4 (the near-infrared wave) and 7 (the medium-infrared wave), respectively, which were calculated according to pre- and post-fire images. In the stratified random sampling design, the dNBR value was classified into two levels according to its histogram: high severity (≥743) and low severity (<743)^29^. In addition, we selected 12 unburned locations (plots), which were classified as mature forest without fire disturbance scattered among fire occurrence region as control. In summary, those samples included no fire (control), 1 year after low intensity fire (OL), 1 year after high intensity fire (OH), 11 years after low intensity fire (EL) and 11 years after high intensity fire (EH). A total of 59 selected samples, 12 from unburned soils and 47 (10 for EL, 13 for EH, 12 for OL and 12 for OH) from burned soils, were analyzed in this study. In each plot (40 m × 40 m), soil was collected from five points (four vertices and the center) at a depth of 0–5 cm and then mixed as one sample. After sampling, the soils were kept in a cooler and shipped refrigerated to the lab. The samples were thoroughly mixed and sieved to remove grassroots and stone, and divided into two parts: one part was stored at 4°C for biogeochemical analysis; the other was stored at −40°C for DNA analysis.

### Soil nutrients and microbial biomass analyses

Soil pH was measured using a pH Meter after shaking a soil water suspension (1:5 wt/vol) for 30 minutes. Soil moisture was measured gravimetrically. Total carbon (TC) and total nitrogen (TN) were determined by dichromate oxidation and titration with ferrous ammonium sulfate^34^. Soil dissolved organic C (DOC) and dissolved total N (DTN) and mineral nitrogen were extracted by adding 50 ml of 0.5 M K_2_SO_4_ to 10 g fresh soil, shaking for 1 h and then vacuum filtering through glass fiber filters (Fisher G4, 1.2 μm pore space). Ammonium (NH_4_^+^) and nitrate (NO_3_^−^) contents in the extracts were determined colourimetrically by automated segmented flow analysis (Bran + Luebbe AAIII, Germany) using the salicylate/dichloroisocyanuric acid and cadmium column/sulphanilamide reduction methods, respectively. DOC and DTN were determined using a TOC-TN analyzer (Shimadzu, Kyoto, Japan). Dissolved organic N (DON) was calculated as follows: DON = DTN − (NH_4_^+^ − N) − (NO_3_^−^ − N). Microbial biomass C (MBC) and biomass N (MBN) were analyzed by the chloroform fumigation and extraction method^35^, and the final values were calculated using 0.35 (k_C_) and 0.4 (k_N_) correction factors^36^.

### Soil DNA extraction

Soil DNA was extracted from the 0.5 g soil after sieving using a FastDNA® SPIN Kit for soil (MP Biomedicals, Santa Ana, CA) according to the manufacturer's instructions. The extracted soil DNA was dissolved in 60 μl TE buffer, quantified by NanoDrop and stored at −20°C.

### Bacterial 16S rRNA genes amplification and 454 Sequencing

An aliquot (50 ng) of purified DNA from each sample were used as template for amplification. The V4–V5 hypervariable regions of the bacterial 16S rRNA genes (*Escherichia coli* positions 515–907) were amplified using the primer set: F515: GTGCCAGCMGCCGCGG with the Roche 454 ‘A' pyrosequencing adapter, and a unique 7 bp barcode sequence, while primer R907: CCGTCAATTCMTTTRAGTTT contained the Roche 454 ‘B' sequencing adapter at the 5′-end of each primer, respectively. The targeted gene region has been shown to be the most appropriate for the accurate phylogenetic reconstruction of bacteria^37^. Each sample was amplified in triplicate with 50 μl reaction under following: 35 cycles of denaturation at 94°C for 45 s, annealing at 55°C for 45 s, and extension at 72°C for 45 s; with a final extension at 72°C for 10 min. PCR products were pooled together and purified by Agarose Gel DNA purification kit (TaKaRa). An equal amount of PCR product for each sample qualitative determination by bioanalyzer (Agilent 2100) and quantitative analysis by NanoDrop was combined in a single tube, and run on a Roche FLX 454 pyrosequencing machine (Roche Diagnostics Corporation, Branford, CT, USA), producing reads from the forward direction F515.

### Processing of pyrosequencing data

Data were processed by the Quantitative Insights Into Microbial Ecology (QIIME) pipeline^38^. Specifically, bacterial sequences with the same barcode were assigned to the same sample after denoising by denoiser v. 0.91^39^. The barcode and primer sequences were removed, and only the first 350 bp after the proximal PCR primer was included for further analysis. Bacterial phylotypes were identified using uclust^40^ and assigned to operational taxonomic units (OTUs, 97% similarity). Representative sequences from each phylotype were aligned using PyNAST^41^,^42^. The taxonomic identity of each phylotype was determined using the ribosomal database project (RDP) Classifier^43^. To correct for survey effort, we used a randomly selected subset of 4,000 sequences per sample to compare relative difference between samples.

### Statistical analysis

Phylogenetic diversities (PD) were estimated by Faith's index^44^, which provides an integrated index of the phylogenetic breadth across taxonomic levels. The relationships between the taxonomic diversity for the group with geochemical features were tested with linear regression analyses using SPSS 17.0 for Windows. The response ratio (RR), calculated using the SAS program (SAS version 9.1. SAS Institute, Cary, North Carolina, USA), was used to analyze the effects of fire on phylogenetic composition and structure of bacterial communities^45^. NMDS (Non-metric multidimensional scaling) using Bray-Curtis dissimilarity and ANOSIM (Analysis of Similarity) based on the OTU table were completed in the *vegan* package (Version 2.0-2) of R v.2.8.1 project (R Development Core Team. Vienna, Austria) to compare community composition in burned and unburned samples. Mantel tests^46^ were performed in the *vegan* package (Version 2.0-2) of R v.2.8.1 project (R Development Core Team. Vienna, Austria) were used to identify environmental factors that significantly correlated with community composition (abundance of OTUs), and the factors that significantly correlated with the bacterial community composition were tested by variance inflation factor (VIF), which is used to judge the colinearity. The VIF value of factors less than 20 were selected to perform canonical correspondence analysis (CCA) in the *vegan* package (Version 2.0-2) of R v.2.8.1 project (R Development Core Team. Vienna, Austria).

## Author Contributions

H.C., X.X. and J.Y. initiated and designed the research; X.X., J.Y. and J.K. collected soil samples; X.X., Y.S. and J.K. performed research; X.X., H.C., H.Z. and Y.S. analyzed the data and wrote the paper. J.Y., J.Z. and X.L. also revised and edited the manuscript.

## Supplementary Material

Supplementary Information supporting information

## Figures and Tables

**Table 1:** The biogeochemical factors that significantly correlated with bacterial communities were listed below. The correlations (r) and significance (P) were determined by Mantel tests between the community composition and environmental variables. SM: soil moisture; TC: total carbon; TN: total nitrogen; C/N ratio: carbon/nitrogen ratio; DOC: dissolved organic carbon; DON: dissolved organic nitrogen; AP: available phosphorus; MBC: microbial biomass carbon; MBN: microbial biomass nitrogen. OYF: 1-year-post-fire; EYF: 11-years-post-fire

	Control		OYF		EYF	
Variables	r	P	r	P	r	P
pH	**0.480**	**0.016**	**0.777**	**0.001**	**0.534**	**0.001**
SM (%)	0.350	0.116	**0.672**	**0.012**	**0.511**	**0.001**
NH_4_^+^ (mg/kg)	0.240	0.113	**0.685**	**0.010**	**0.221**	**0.028**
C/N Ratio	0.250	0.113	**0.681**	**0.038**	**0.229**	**0.026**
NO_3_^−^ (mg/kg)	0.182	0.082	0.164	0.117	**0.507**	**0.006**
TN (%)	0.181	0.772	0.153	0.127	0.022	0.378
TC (%)	0.193	0.767	0.107	0.177	0.267	0.087
AP (mg/kg)	0.292	0.667	0.137	0.123	0.167	0.107
DOC (mg/kg)	0.166	0.672	0.030	0.407	0.166	0.084
DON (mg/kg)	0.147	0.683	0.078	0.202	0.019	0.447
MBC (mg/kg)	0.149	0.767	0.079	0.693	0.129	0.139
MBN (mg/kg)	0.102	0.562	0.103	0.746	0.057	0.282
Elevation (m)	0.196	0.235	0.112	0.463	0.128	0.368
