# Supplementary material for: Rapid recovery of soil bacterial communities after wildfire in a Chinese boreal forest
Source: Sci Rep. 2014 Jan 23;4:3829. doi: 10.1038/srep03829 (PMC3899593; doi:10.1038/srep03829)
Supplement: Supplementary Information — supporting information [file srep03829-s1.doc]

Title: Rapid recovery of soil bacterial communities after wildfire in a Chinese boreal forest

Authors: Xingjia Xiang^1,3,+^, Yu Shi^1,3,+^, Jian Yang^2^, Jianjian Kong^2,3^, Xiangui Lin^1^, Huayong Zhang^1^, Jun Zeng^1^, Haiyan Chu^1,*^

^1^ State Key Laboratory of Soil and Sustainable Agriculture, Institute of Soil Science, Chinese Academy of Sciences, East Beijing Road 71, Nanjing 210008, China

^2^State Key Laboratory of Forest and Soil Ecology, Institute of Applied Ecology, Chinese Academy of Sciences, Wenhua Road 72, Shenyang 110164, China

^3^University of the Chinese Academy of Sciences, Beijing 100049, China

^+^The authors contributed equally to this work

***Corresponding author:**

Haiyan Chu, Tel.: 86-25-86881356

E-mail: [hychu@issas.ac.cn](mailto:hychu@issas.ac.cn)

Table S1: Summary of the main characteristics of sampling sites in the Greater Khingan Mountains. The values in brackets represent the standard deviation of the mean. Different letters represent significant differences from Tukey’s HSD comparisons (P < 0.05). OL: one year after low intensity fire; OH: one year after high intensity fire; EL: 11 years after low intensity fire; EH: 11 years after high intensity fire. SM: soil moisture; TC: total carbon; TN: total nitrogen; C/N: carbon/nitrogen; DOC: dissolved organic carbon; DON: dissolved organic nitrogen; MBC: microbial biomass carbon; MBN: microbial biomass nitrogen.

Table S2: Relative average abundances of phyla classified with RDPII taxonomy across all soils and soils grouped into different treatments (values represent % of total sequences). Asterisks indicate sequences classified to the domain Bacteria, but not to a specific phylum. The “0.00” value means less than 0.01. OL: one year after low intensity fire; OH: one year after high intensity fire; EL: 11 years after low intensity fire; EH: 11 years after high intensity fire.

Table S3: Differences of bacterial communities across the different groups examined by the dissimilarity test of ANOSIM. Pearson correlations coefficients (r) are shown with associated Bonferroni-corrected P-value. P < 0.05, significant convention. OYF: 1 year post fire; EYF: 11 years post fire.

Table S4: Correlations between the relative abundances of the dominant bacterial phyla and soil characteristics and altitude by linear regression analyses. Pearson correlations coefficients (r) are shown for each taxon with associated Bonferroni-corrected P-value. SM: soil moisture; TC: total carbon; TN: total nitrogen; C/N ratio: carbon/nitrogen ratio; AP: available phosphorus; DOC: dissolved organic carbon; DON: dissolved organic nitrogen; MBC: microbial biomass carbon; MBN: microbial biomass nitrogen.

*: P < 0.05; **: P < 0.01, significant convention

Table S5: Pearson correlations (R) between bacterial diversity (Faith’s PD, OTUs) and soil and site characteristics by linear regression analyses. SM: soil moisture; TC: total carbon; TN: total nitrogen; C/N ratio: carbon/nitrogen ratio; AP: available phosphorus; DOC: dissolved organic carbon; DON: dissolved organic nitrogen; MBC: microbial biomass carbon; MBN: microbial biomass nitrogen.

*: P < 0.05, significant convention


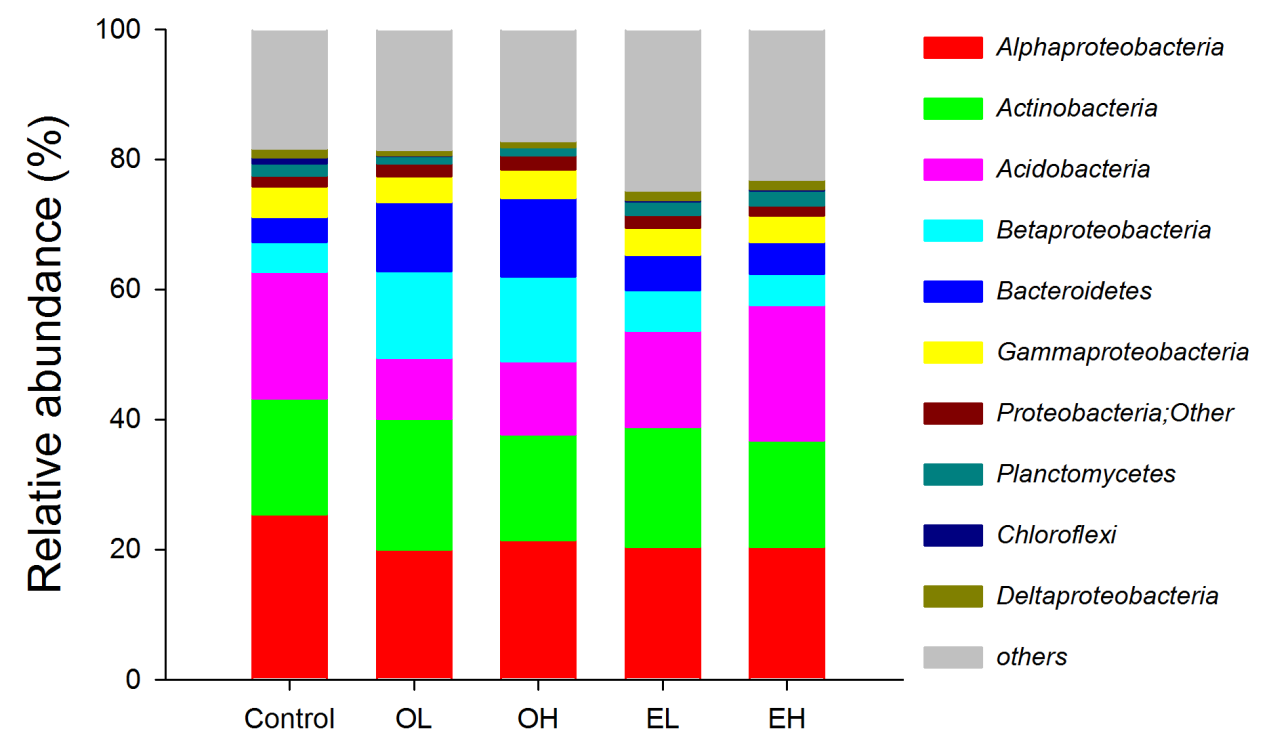
Figure S1: Relative abundances of the dominant bacterial phyla in soils. Relative abundances are based on the proportional frequencies of DNA sequences that could be classified at the phylum level. OL: one year after low intensity fire; OH: one year after high intensity fire; EL: 11 years after low intensity fire; EH: 11 years after high intensity fire.

Figure S2: OTUs that exhibited significant changes in abundance at one year after low intensity fire. Significance was determined using response ratio methods at a 95% CI (confidence interval).
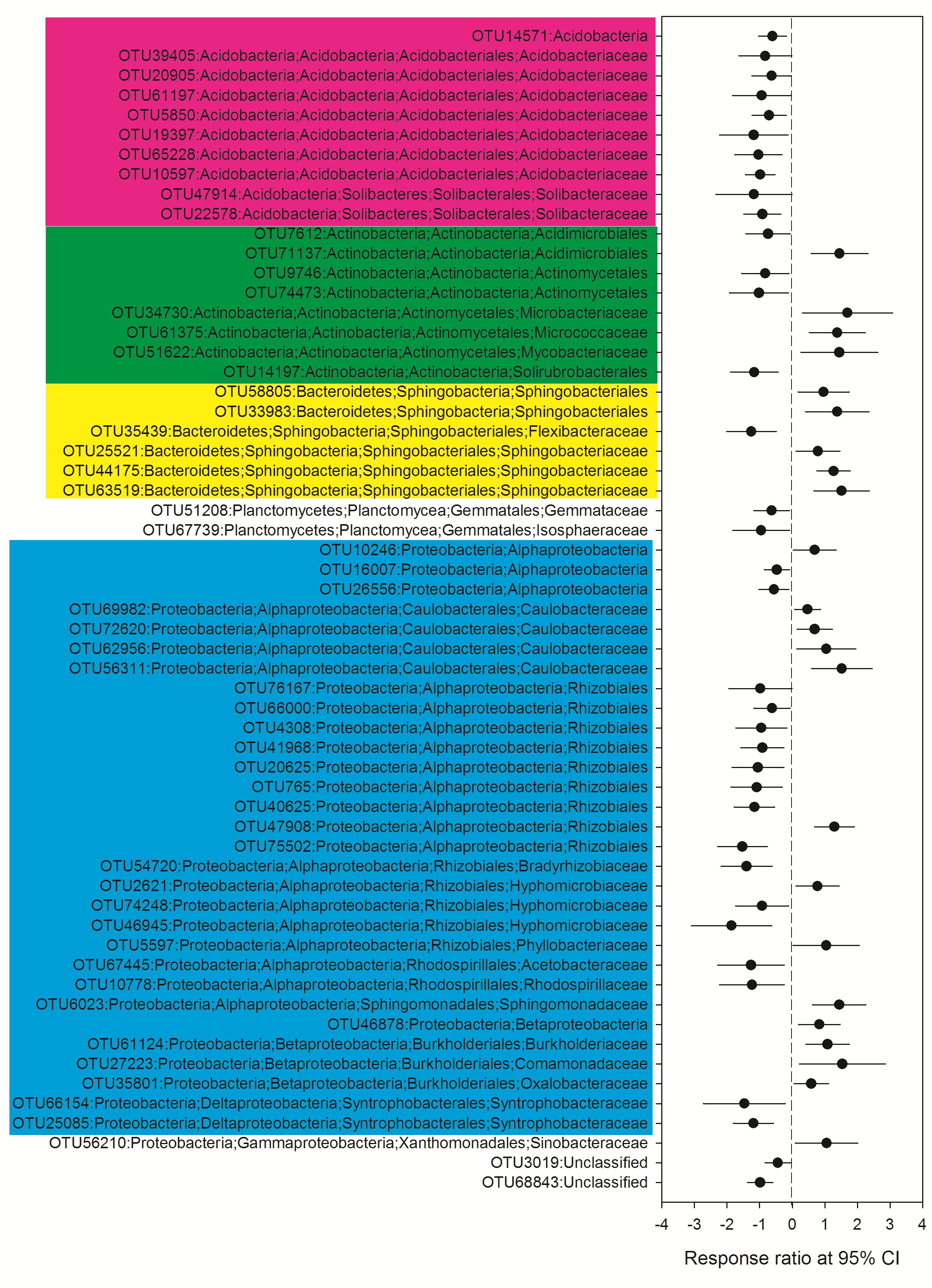


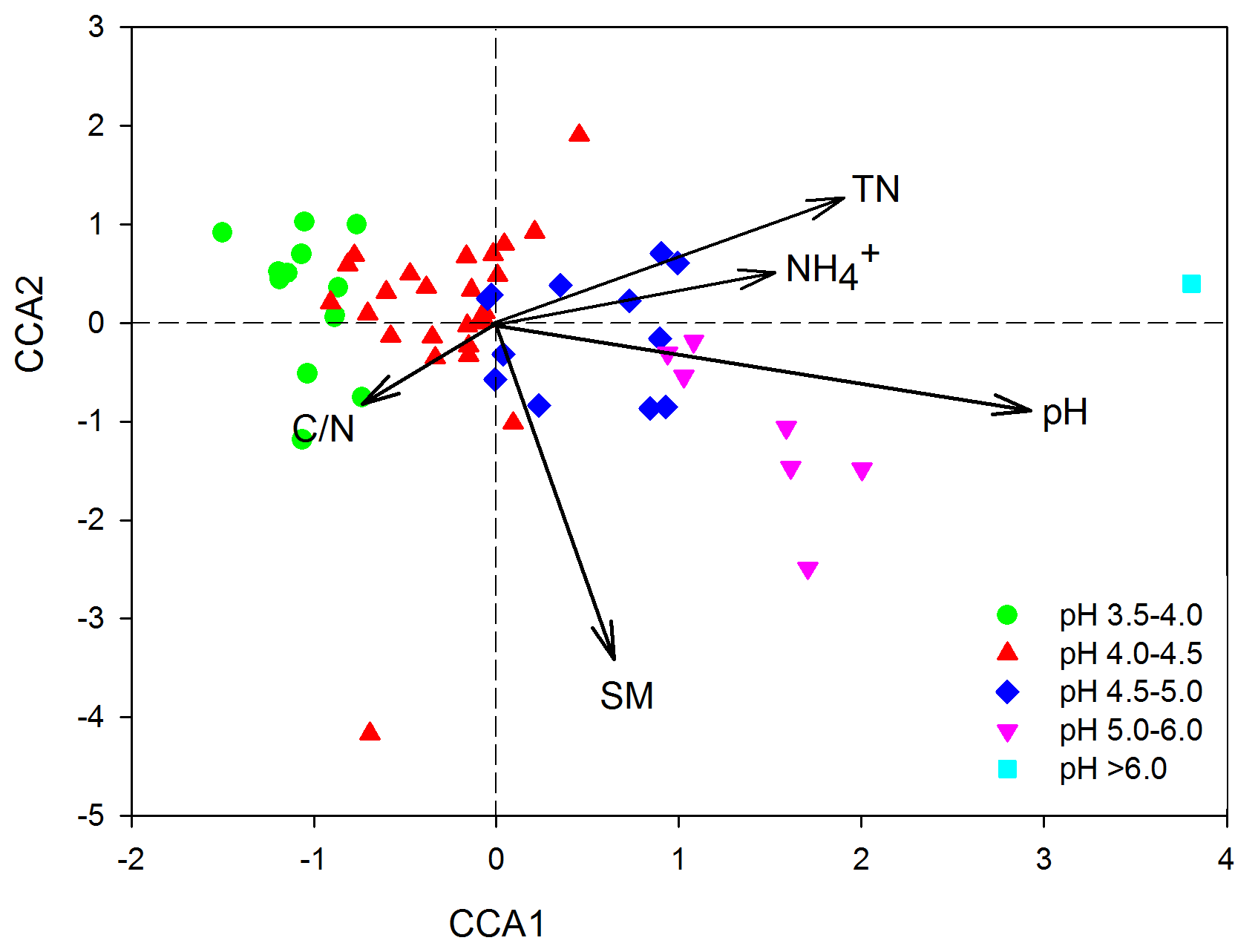
Figure S3: Canonical correspondence analysis (CCA) of the bacterial communities was completed in the *vegan* package of R v.2.8.1 project. SM: soil moisture; TN: total nitrogen; C/N ratio: carbon/nitrogen ratio.


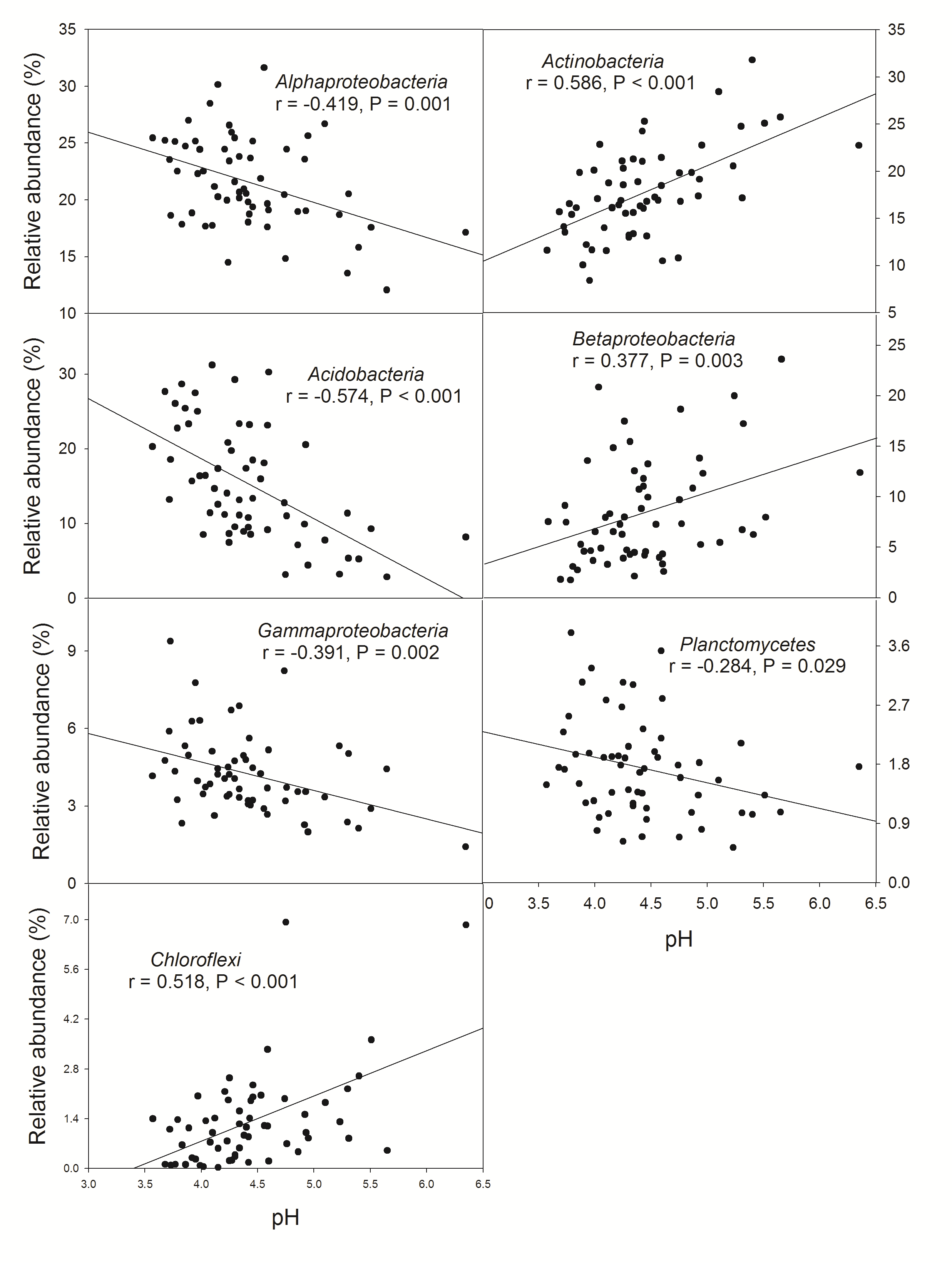
Figure S4: The relationships between relative abundances of dominant bacterial groups and soil pH. Linear regressions were used to test the correlation between the taxa’s relative abundances and pH. P < 0.05, significant convention.
